# Genetic engineering in organoids

**DOI:** 10.1007/s00109-020-02029-z

**Published:** 2021-01-18

**Authors:** Isaree Teriyapirom, Andreia S. Batista-Rocha, Bon-Kyoung Koo

**Affiliations:** Institute of Molecular Biotechnology of the Austrian Academy of Sciences (IMBA), Vienna Biocenter (VBC), Dr. Bohr-Gasse 3, 1030 Vienna, Austria

**Keywords:** 3D Organoids, Organoid Genetics, Pluripotent Stem Cells, Adult Stem Cells, Genetic Engineering, CRISPR/Cas9, Disease Modeling

## Abstract

Three-dimensional organoids have been widely used for developmental and disease modeling. Organoids are derived from both adult and pluripotent stem cells. Various types are available for mimicking almost all major organs and tissues in the mouse and human. While culture protocols for stepwise differentiation and long-term expansion are well established, methods for genetic manipulation in organoids still need further standardization. In this review, we summarized different methods for organoid genetics and provide the pros and cons of each method for designing an optimal strategy.

## Introduction

Organoids are three-dimensional (3D) in vitro cultures derived from stem or progenitor cells, which can recapitulate the variety of cell types, architectural organization and function of their in vivo tissue counterparts [17, 64]. The first attempt of generating organs in vitro began in 1907 when Wilson demonstrated that dissociated sponge cells could reaggregate and self-organize to reform the whole organism [117]. Current attempts to generate organ-specific models grew from the work of Sasai and colleagues, who showed that three dimensional (3D) cerebral cortical tissue could be generated in vitro from pluripotent stem cells [26], as well as from the work of Clevers and colleagues, who generated gut organoids from adult intestinal stem cells [95]. These studies led to the classification of organoids into two main categories: pluripotent stem cell (PSC)-derived organoids and adult stem cell (AdSC)-derived organoids.

As there are already multiple reviews comparing these two categories [17, 43, 64], this chapter will provide only a short summary of the major distinguishing factors between PSC- and AdSC-derived organoids. In principle, PSC-derived organoids are grown from either embryonic stem cells (ESCs) or induced pluripotent stem cells (iPSCs), which we will collectively refer to as PSCs. These cells are first cultured in suspension in a defined medium to promote cell aggregation and directed differentiation [43]. Cell clusters are then embedded in a matrix that provides structural support, allowing the cells to organize into structures resembling the endogenous tissue. PSC-derived organoids can contain different cell types originating from the different germ layers (ectoderm, mesoderm, and endoderm). Since the first 3D cultures of the cerebral cortex [26], organoid differentiation protocols have been developed for generating models of various other tissues, based on the presence of specific signaling factors in the medium. Established murine PSC-derived organoids now include models of the optic cup [27], pituitary gland [105], inner ear [59], and thyroid gland [4, 63]. Human PSC-derived organoids include models of the brain [65], kidney [77, 106], small intestine [102], stomach [73], lung [25], liver [107], colon [79], and mammary gland [88].

AdSC-derived organoids, on the other hand, do not require directed differentiation, as they are grown from tissue-resident adult stem cells in a similar process to that used for the sponge cell reaggregation [117]. AdSCs are first extracted from the organ by tissue dissociation, then directed to form organoids in medium that supports their stem cell activity with an optimal growth factor combination. Examples of mouse AdSC-derived organoid cultures include the intestine [95], stomach [8, 104], liver [15, 41, 42, 45, 83], pancreas [15, 44], lung [67], endometrium [14], salivary gland [81], and taste bud [90]. Human AdSC-derived organoids have also been developed for the intestine [51, 96], liver [15, 41, 42], pancreas [15], endometrium [14, 109], fallopian tube [55], and prostate [53]. While generating organoids from AdSCs requires less time than from PSCs, the number of different cell types that can be generated from AdSCs is limited, as AdSC-derived organoids often only contain epithelial cells [43]. For this reason, they are useful for studying epithelial tissue maintenance and regeneration but not suitable for studies involving the interaction between different cell types, e.g., immune-epithelial interaction.

Since their development, organoids quickly became a popular model by bridging the gap between in vivo animal models, which are time-consuming to generate and costly to maintain, and in vitro two-dimensional cell culture systems, which lack 3D tissue organization and often contain cancer-associated genetic alterations. 3D organoid systems have been used for studying organ development [65] and host-pathogen interactions [20, 87]. They can also be used for disease modeling and therapy development, e.g., by using cancer and diseased tissues as starting materials for organoid formation [10, 11, 34, 65, 66, 96, 110]. Despite all these achievements, the ability to generate, repair, or introduce specific genetic mutations was needed for modeling monogenic disease and cancer, as well as for genome-wide screening and establishing reporter organoids.

## Genetic engineering methods

There are currently multiple methods of genetic engineering that have been employed in organoids, opening a new field of research—organoid genetics. These methods enable specific modifications of the genomic DNA sequence. If the modifications are introduced in a coding sequence, they can lead to a specific change in the target protein, which can provide insight into the biological role of a specific residue or the protein itself. This process requires consideration of two major points: the genetic tools and the method of delivering them into the target cells.

### Methods for delivery

**Fig. 1:**
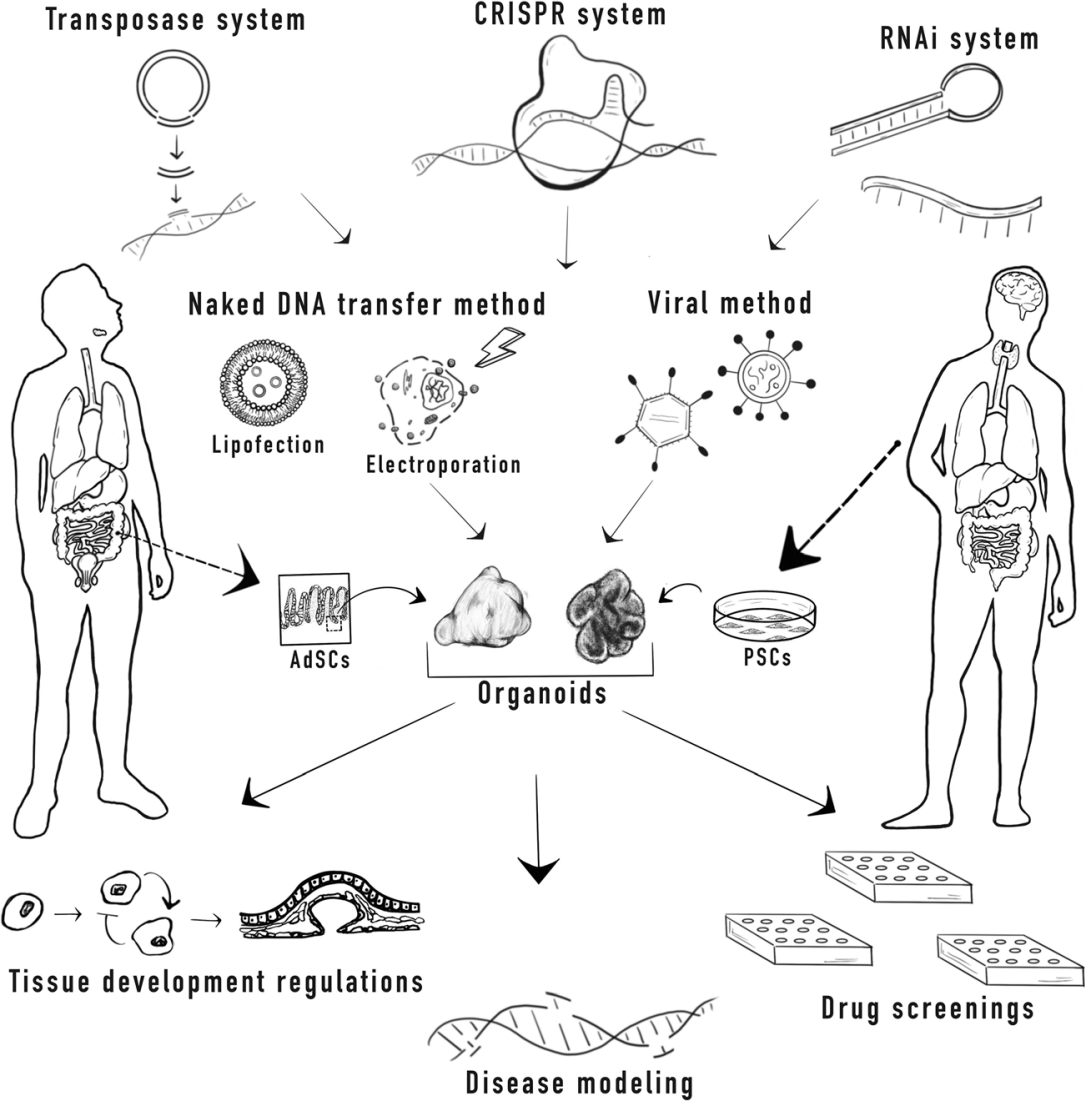
Methods of generating organoids and genetic engineering with their possible applications. Organoids can be generated either from adult stem cells (AdSCs) or pluripotent stem cells (PSCs). AdSCs, extracted from the tissue of origin, can be cultured with the proper conditions to give rise to organoids that mimic the organ they derive from. PSC-derived organoids are grown from cell line of induced pluripotency or embryonic stem cells. Depicted on the left and right human figures are the types of organoids which have been generated with AdSCs or PSCs, respectively. Organoids can be modified with different genetic engineering methods such as CRISPR/Cas, transposase, or RNAi. These tools could be delivered with a non-viral approach such as lipofection or electroporation, or with a viral approach utilizing retrovirus, lentivirus, or adenovirus. The genetically edited organoids can be further utilized for various applications/fields of study including biological developmental models and translational/precision medicine

There are currently two common methods of introducing gene-editing components into organoids: viral (e.g., retro/lentiviral or adenoviral transduction) and non-viral using naked DNA transfer (Fig. 1). Each method has its advantages and disadvantages, which will be briefly discussed here and summarized in Table 1. Choosing the appropriate delivery system requires consideration of the properties of the target cells, the size of the DNA fragment, and the required duration of gene expression.

**Table 1:** Pros and cons of different methods of delivery

	Viral			Non-Viral	
	Retrovirus	Lentivirus	Adenovirus	Electroporation	Lipofection
Pros	Stable integration	Stable integration Can infect non-dividing cells	Infect dividing and non-dividing cells (Transient integration) Easy to achieve high viral titer	Efficient for any cell types and living organisms Can introduce large constructs Low DNA requirement	Simple to use Efficient in many cell types
Cons	Cannot infect non-dividing cells Can induce immune response Transgene size limited to 8 kb Time-consuming for virus production Issues with biosafety and mutagenesis	Transgene size limited to 8 kb Time-consuming virus production Issues with biosafety and mutagenesis	Transgene can be lost over divisions Issues with biosafety and mutagenesis	Costly Require extensive optimization Potential cell damage/nonspecific transport to cells	Transient transgene expression May affect cell survival

Retro- and lentiviral transfections utilize the viral machinery to induce stable integration of foreign genetic sequences whose expression can be consistently passed on to progenies [62]. However, retroviruses rely on the host cell cycle to integrate genetic information into the genome, thus cannot infect terminally differentiated, non-dividing cells. Furthermore, retrovirus infection requires high viral titer and can induce immune responses that may reduce the efficiency of genome integration [92, 101]. Lentiviruses have an adaptation that circumvents this limitation, and are thus commonly used for cells that are difficult to infect, such as immune cells or non-dividing cells [21]. However, with both retro- and lentiviruses, integration preferentially occurs in transcriptionally active sites, which can adversely affect the expression of host genes. Moreover, both viral vectors can only accommodate a maximum DNA insert of about 8 kb, which covers most cDNAs, but not all [21, 46].

The adenoviral method avoids permanent integration by remaining episomal after transfection and is effective in both dividing and non-dividing cells [101]. It is also easy to generate high virus titers for higher expression of the introduced transgene. However, due to the lack of genomic integration, the introduced gene can be lost over multiple rounds of host cell division [114, 115].

Lastly, non-viral naked DNA transfer generally involves one of two delivery approaches: electroporation or lipofection. Electroporation utilizes electrical pulses to transiently create openings in the cell membrane, allowing foreign DNA to enter the cell [32]. This method is usually efficient for many cell types and even in living organisms, and can also easily introduce large constructs into the cell. Nevertheless, electroporation requires a relatively expensive device and extensive pilot testing as the optimal parameters vary significantly for each device and cell type. Lipofection utilizes Lipofectamine or related lipid molecules that can form liposomes, encapsulating DNA and introducing it into the cell [97]. This method is relatively simple and usually efficient enough in many cells. However, the transgene expression is normally transient, and lipofection may affect cell survival.

### Tools for genetic engineering

After deciding on a method of genetic delivery, it is important to consider the method of genetic editing. As there are many reviews comparing the different tools for genetic engineering [19, 56, 57, 120], we will only present a short summary of important methods that have been used to genetically modify organoids: RNA interference (RNAi), CRISPR/Cas9, retro/lentiviruses, and transposons (Fig. 1 and Table 2).

**Table 2:** Pros and cons of gene-editing techniques

	RNA interference (RNAi) system	Transposon-based system	CRISPR/Cas9
Pros	Effective in all mammalian somatic cells No prior genetic manipulation needed Multiplexing possible	Stable integration for long-term expression	Introduce specific modification to target sequence Multiplexing possible Ease of scalability
Cons	Knockdown system only Lower efficacy Prone to off-target effects	Random insertion can disrupt transcriptionally active genes Difficult to perform on large scale	Susceptible to immune reaction Possible off-target effects

The RNAi system utilizes the cell’s own machinery to silence expression of specific genes. In this system, synthesized RNAi sequences, either short-hairpin RNAs (shRNAs) or short-interfering RNAs (siRNAs), form complementary pairs with the mRNAs of the target gene to promote degradation or translational silencing and thereby suppress the protein expression of the target mRNA. This method is effective in all mammalian somatic cells and no prior genetic manipulation is necessary [28, 39]. shRNAs can be delivered into cells with various vectors such as retro-/lentiviruses, adenoviruses, plasmids, and transposons. [120]. However, RNAi is only a knockdown system, has lower efficacy, and is prone to off-target effects.

A useful choice for stable gene expression is transposon-based systems, e.g., PiggyBac and Sleeping Beauty, which can stably introduce the gene of interest into the host genome for long-term expression. Both PiggyBac and Sleeping Beauty use the “cut-and-paste” mechanism to “cut” the genetic sequence flanked by a specific terminal inverted repeat from one locus and “paste” it into another [46]. However, this random insertion sometimes occurs in an active gene, which can lead to unexpected effects on the host cell.

Since 2012, clustered regularly interspaced short palindromic repeats (CRISPR)/CRISPR-associated (Cas) systems have been widely adapted for sequence-specific editing in both prokaryotic and eukaryotic cells in vitro [16, 18, 35, 50, 71]. The system was first discovered in bacteria, giving them adaptive resistance to bacteriophage infections [9]. The CRISPR/Cas9 system was then further engineered with two components: Cas9 endonuclease and single-guide RNA (sgRNA or gRNA), where a spacer sequence binds to a complementary sequence of DNA (protospacer sequence) and guides Cas9 to a specific target. A DNA target containing both the protospacer sequence and the protospacer adjacent motif (PAM) forms a target for the Cas9; gRNA complex to introduce a double-strand break (DSB). The PAM sequence differs for the different Cas9 and Cas12a (Cpf1) endonucleases derived from different bacteria species, thus enabling a broad range of applications [18, 89, 121]. Following cleavage by the Cas9 nuclease, the DSB can be repaired by either homology-directed repair (HDR), which requires a template for precise, high-fidelity repair or by non-homologous end joining (NHEJ), where the blunt ends are re-ligated together [13]. Repair by HDR following a supplied template allows researchers to introduce specific sequence changes into target genes [33]. However, this process is inefficient and requires the cell to be in S phase of the cell cycle for the repair to occur [47]. Template plasmid must also be cloned with homology arms specific to each gene, thus increasing the work effort. Alternatively, DNA repair can occur by nucleotide insertions or deletions introduced by NHEJ which generate frameshifts mutations, leading to inactivation of the target gene. As NHEJ is often viewed as error-prone, it is not used for precise targeted mutation. However, recent work by Artegiani et al. [6] adapted the NHEJ for generation of fast knock-ins in various human organoids. This method removes the effort required for homology arm cloning as knock-in DNA is cloned into a self-cleaving plasmid containing a non-human sequence which is recognized by sgRNA. The authors could show more efficient knock-in generation as compared to HDR even with TP53 inhibition which was suggested to improve HDR efficiency in human pluripotent stem cells [6, 48].

Further advances in CRISPR/Cas9 development also target increasing efficiency of different Cas enzymes to detect a broader PAM sequence range [41, 42] or nearly remove the PAM sequence constraint completely [113]. Modification of the Cas9 endonuclease by fusing inactivated Cas9 nickase to cytidine deaminase to generate base editors [36, 40, 60]. This introduced new tools to generate precise base changes in organoids [37, 112].

Combining organoid technology with the various genetic editing techniques provides a new platform for organoid genetics and organoid-based disease modeling. The following chapter provides further details on the applications of gene editing with the CRISPR/Cas9 system in various AdSC- and PSC-derived organoids.

## Editing in adult stem cell-derived organoids

**Fig. 2:**
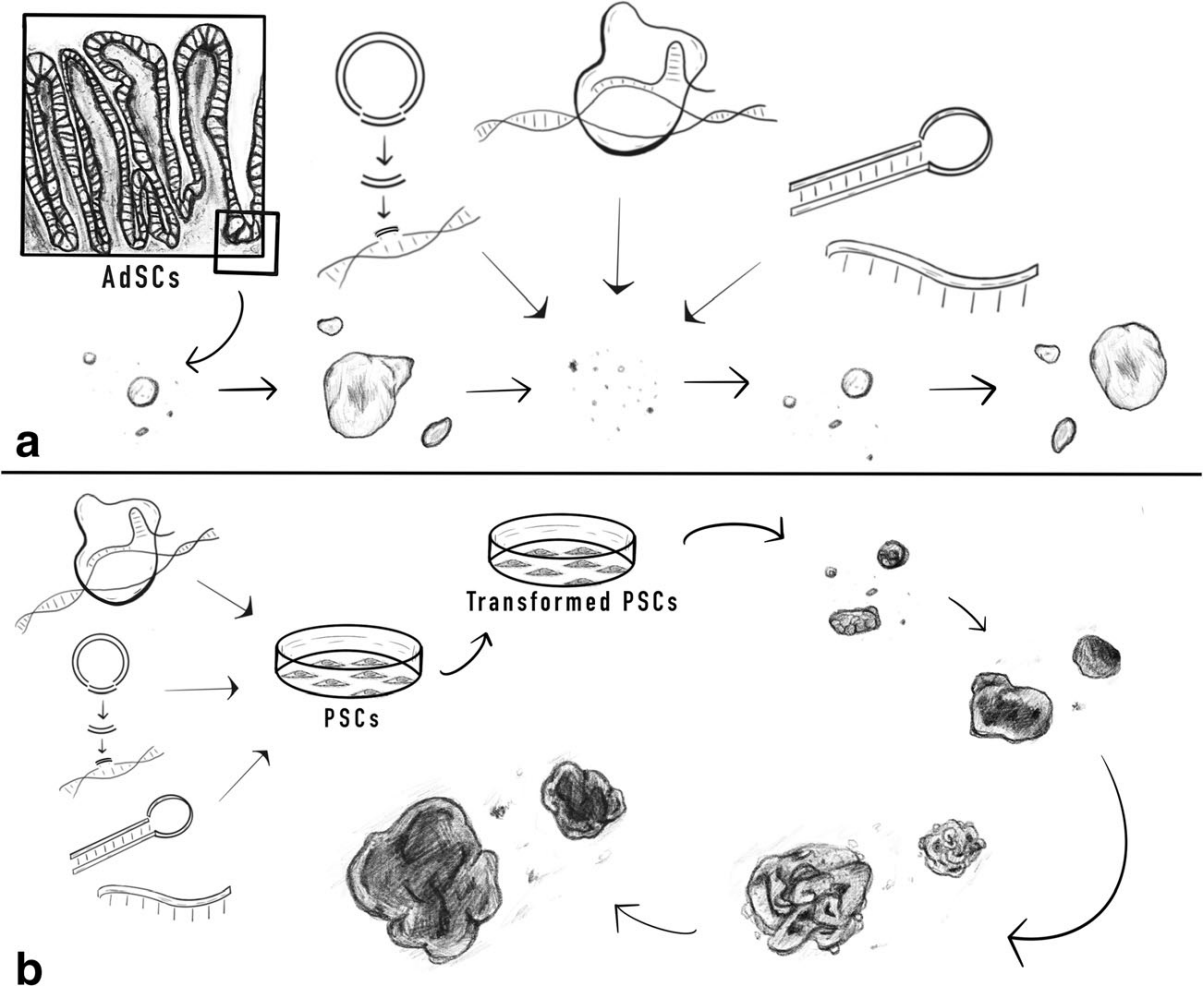
Comparison of genetic engineering in AdSCs and PSCs. **a** With the AdSC-derived organoids, it is necessary to first establish the organoids from the tissue-resident stem cells and culturing them as unedited organoids, allowing them to stabilize under in vitro conditions. To edit these organoids, we must dissociate them to single-cell state in order to introduce the gene-editing tools efficiently. Single cells will then grow back to re-form organoids which can be maintained in culture or frozen for long-term. **b** PSCs-derived organoids, on the other hand, can be genetically modified prior to the organoid formation. Gene-editing tools can be introduced to the PSCs directly; then, the PSCs could be differentiated to form genetically modified organoids. After the organoid is formed, it cannot be dissociated again without losing its structural integrity and function. This figure represents an example of the different stages for brain organoids formation

The establishment of AdSC-derived organoids depends on the proliferation and differentiation ability of the AdSCs. To establish organoids of this type, AdSCs are isolated from the resident tissue and cultured in a laminin-rich matrix with the required growth factors that mimic the niche environment required for maintenance of AdSCs and differentiation to the organoid structure. Because AdSCs are isolated directly from tissues, one method of obtaining a genetically engineered organoid line is to isolate AdSCs from a genetically engineered animal model with the desired genetic mutation. In 2012, Koo et al. showed that deletion of RNF43 and ZNRF3 from LGR5+ stem cells in the intestinal epithelial compartment induces adenoma growth containing Paneth cells and undifferentiated LGR5+ cells. Organoids containing the double mutation could be derived from this adenoma: they mimicked the same effect as in vivo and could be used for drug testing [61]. However, generating a genetically mutant mouse line or acquiring patient-derived tissues with desired mutations to start organoid culture can be difficult, time-consuming, and costly [23]. Thus, it would be advantageous to be able to edit gene expression in organoids directly (Fig. 2a).

### Gastrointestinal tract organoids

In 2013, Schwank et al. utilized a transient CRISPR/Cas9 targeting system mediated by lipofection to demonstrate that CRISPR/Cas9 could be applied in organoids for genetic knock-out or mutation repair [98]. First, the authors induced a frameshift mutation in the *APC* gene of mouse and human organoids. As APC is a well-established tumor suppressor and negative regulator of WNT signaling, the targeted organoids displayed WNT signaling-independent growth. With the application of the tool established, the team then repaired mutations in the gene coding for cystic fibrosis transmembrane conductor receptor (CFTR) in colonic organoids derived from cystic fibrosis patients. The targeted organoids showed restored swelling in response to forskolin, indicating successful repair of the disease-causing deletion mutation in the *CFTR* locus. The results show the potential usefulness of CRISPR/Cas9 editing in organoid research and in correcting monogenic disorders [98].

One focus of gene editing in organoids has been to model the effect of oncogenes in tumor evolution. Downregulation of tumor suppressor *Tgfbr2* expression in gastric organoids through retroviral delivery of *Tgfbr2* shRNA produced various gastric cancer subtypes [80]. To further understand the complex interaction of oncogenes in tumor initiation, mouse colon, stomach, and pancreatic organoids were grown from conditional *Apc* knockout mice and then manipulated to induce overexpression of mutant *Kras* or downregulation of *Tp53* or *Smad4* expression using shRNA. While pancreatic and gastric organoids showed abnormal growth in response to either single or combinatorial mutations, colonic organoids required combinatorial mutations to initiate transformation [68]. The coupling of genetic manipulation and organoid modeling was thus able to confirm the multi-hit model postulated for various cancer types.

The multi-hit oncogenesis model was further explored by two independent studies that used CRISPR/Cas9 to introduce mutations into colonic organoids [24, 72]. Both studies targeted *APC*, *SMAD4*, *TP53*, and *KRAS*, with the study led by Matano et al. targeting *PI3KCA* as well. CRISPR/Cas9 editing induced a gain-of-function mutation in *KRAS* and a loss-of-function in all the other genes. Mutated organoids all showed independence from different growth factors with varying degrees of invasive behavior when transplanted, depending on the mutation(s) induced. Drost et al. also showed that sequential oncogene modifications of *APC* and *TP53* were sufficient for independence from growth factors and development of aneuploidy.

Furthermore, CRISPR/Cas9 editing in organoids has been useful for modeling diseases that could not previously be recapitulated in vitro. Sessile serrated adenomas (SSAs) are premalignant lesions of the colon, which differ both histologically and molecularly from lesions associated with mutations in the *APC* gene, even though both are able to drive colorectal cancer development [108]. Following analysis of polyps from SSA patients, the activating *BRAF* proto-oncogene with *V600E* mutation was commonly found in association with the disease [103]. By introducing the *BRAF*^*V600E*^ mutation into wild-type colorectal organoids, Fessler et al. obtained organoids that grew independently from TGF-β signaling and showed epithelial-mesenchymal transition, a phenotype associated with early oncogenesis [30].

Genetic editing in organoids can also be used in screening for efficient drug responses. Verissimo and colleagues used colorectal organoids harboring different RAS mutations to test how they affect the response to EGFR and MEK inhibitors [111]. While wild-type organoids showed sensitivity to the inhibitors, organoids with oncogenic KRAS mutations introduced by CRISPR/Cas9 displayed reduced sensitivity to the drugs. In the mutant organoids, the drugs induced cell-cycle arrest rather than cell death, demonstrating the potential of using CRISPR/Cas9-edited organoid libraries for large-scale screens.

However, to study the effect of multiple mutations simultaneously using a CRISPR/Cas9 knockout screen, cloning and delivery of multiple gRNAs are required, thus minimizing the efficiency of the process in a large-scale study. The development of a new tool for multiplexing gRNA expression in a concatemer vector generated through the design of specific gRNA overhangs allows one-step cloning of up to four gene knockouts simultaneously [3]. As a proof of concept, the authors induced simultaneous knockouts of genes involved in *WNT* signaling regulation and their paralogues in small intestinal organoids and demonstrated that the organoids became WNT independent. This method could be useful for performing screens with multiple gene targets in oncogenic or disease modeling studies.

More recently, human colon-derived organoids have been edited to model traditional serrated adenoma (TSA) [54]. This rare subtype of colonic serrated adenomas is characterized by distinct serration morphology, villiform structures, and ectopic crypt formation. Using CRISPR/Cas9 introduced by electroporation in wild-type and *TP53*-knockout human colonic organoids, the authors knocked in a *BRAF* mutation, overexpressed the *GREM1* gene, and generated long-range gene fusions. Following transplantation, organoids expressing mutant BRAF and GREM1 displayed phenotypes similar to those seen in TSA patients.

### Liver organoids

The liver contains two epithelial cell types: hepatocytes and ductal cells. Expression of *LGR5*, which marks adult stem cells in the stomach, intestine, and colon, is barely present in homeostatic, healthy liver. However, upon injury, Lgr5+ cells appear around the bile duct to replenish the damaged tissue [45]. Because of this proliferative capacity, Lgr5+ cells were also used to establish liver organoids, which could be transplanted to rescue fumarylacetonacetate hydrolase (FAH)-deficient mice from liver failure upon withdrawal of nitisinone (NTBC) [7].

In 2016, Broutier et al. published a protocol detailing methods for genetic manipulation of liver and pancreatic organoids. The established tool utilized stably integrated retroviral transduction or transiently expressed liposomal transfection coupled with CRISPR/Cas9 editing. Editing efficiency was determined by detection of a fluorescent reporter or expression of a drug selection marker [15]. However, these methods required organoid dissociation into single cells, thus lowering the editing efficiency overall. Liver organoids can also be edited using adeno-associated virus vectors [116]. Using the adapted AAV-DJ vector expressing human HNF4α, a master regulator inducing hepatocyte differentiation, the authors showed increased hepatocyte formation in liver organoids compared to wild-type controls.

### Mammary epithelial organoids

The existence of tissue stem/progenitor cells in the mammary gland is suggested by the multiple cycles of tissue remodeling during the menstrual cycle and pregnancy. The first human mammary gland organoid culture was reported by Linnemann and colleagues in 2015 [69]. Since then, there have been many refinements of mammary gland organoid culture to improve recapitulation of in vivo tissue architecture and function [49, 100].

Recently, Dekkers and colleagues showed that it is possible to use CRISPR/Cas9 editing in mammary epithelial organoids derived from human reduction mammoplasty patients in order to model the clonal evolution in breast cancer development [22]. Using mammary epithelial cells sorted from normal reduction mammoplasties, the team generated human mammary epithelial organoids. Targeted knockout of four tumor suppressor genes, *TP53*, *PTEN*, *RB1*, and *NF1*, induced long-term culturability and tumor formation upon murine transplantation. This work suggests a potential use of CRISPR/Cas9 editing as a technique for modeling mammary epithelial-associated diseases.

## Editing in pluripotent stem cell-derived organoids

Pluripotent stem cells can give rise to cells from all three germ layers in vitro, given the appropriate culture conditions. For this reason, organoids generated from PSCs often consist of cells derived from more than one germ layer, allowing the study of interaction between various cell types and providing a model that more closely mimics endogenous organs [79, 102]. Furthermore, establishing gene-edited organoids from PSCs is relatively more straightforward than from AdSCs, as editing can be done directly in PSCs prior to organoid differentiation as discussed below (Fig. 2b). This circumvents the need to dissociate organoids for transfection of the genome-editing machinery, which may reduce the overall efficiency of organoid editing [23].

### Brain organoids

The brain is one of the most complex organs in the vertebrate body. Studies to understand the human brain had been limited by the accessibility to brain tissue and ethical constraints. However, following initial reports by the group of Yoshiki Sasai, brain research has benefited from the development of 3D in vitro models of different specific regions of the brain, including the cortex [26], pituitary gland [105], cerebellum [78], hippocampus [52], and even whole brain organoids [65]. These models provide a platform for understanding factors regulating normal brain development.

As brain organoids are solely derived from PSCs, disease modeling in brain organoids has relied heavily on human iPSCs derived from patients, with or without genetic editing of the iPSCs prior to organoid differentiation. For example, Sandhoff disease, a lysosomal storage disorder caused by a mutation in *HEXB*, leading to neurodegeneration and early childhood death, was modeled using cerebral organoids developed from patient-derived human iPSCs [2]. CRISPR/Cas9-edited correction of the loss-of-function mutation in human iPSCs produced disease-free organoids, indicating that CRISPR/Cas9 gene editing could be used for testing the importance of disease-causing mutations. A glioblastoma cancer model was also developed by introducing an activating *HRAS* oncogenic mutation into the *TP53* locus of human PSCs, knocking out the *TP53* allele while introducing the *HRAS* oncogene [82]. In addition, human iPSCs were edited to introduce the *LRRK*^*G2019S*^ mutation and differentiated to form midbrain organoids that display features of Parkinson’s disease [58].

One potential disadvantage of introducing genetic modifications into PSCs is that it results in the generation of organoids containing only mutant cells, thus limiting the interaction between wild-type cells and mutants that would normally exist in endogenous diseased tissues. A new method of introducing oncogenic mutations directly into neuroepithelial cells, rather than into PSCs prior to neural induction, using CRISPR/Cas9 to induce tumor suppressor knockout together with Sleeping Beauty transposons to induce oncogene expression, generated organoids that more closely mimic the in vivo tumor organization, containing both transformed and non-transformed cells in close proximity [12]. This model is valuable for tumor studies as well as for drug screening, as it provides a platform that enables study of the wild-type cells in the same system.

### Gastrointestinal tract organoids

While gastrointestinal tract organoids such as gastric, intestinal, and colorectal organoids have been successfully generated from tissue-resident stem cells [10, 51, 96], it is also possible to derive organoids of these organs from PSCs [73, 74, 79, 102]. This method provides a useful resource for performing genetic studies of human diseases. Woo and colleagues used the CRISPR/Cas9 system to generate iPSCs with a mutation in the *DKC1* gene, which encodes DYSKERIN, a protein essential in telomere maintenance [118]. Patients with dyskeratotis congenita (DC) often have a mutation in the *DKC1* gene leading to accelerated degeneration of highly-proliferative tissues such as the epidermis, the gastrointestinal tract, and the hematopoietic system [5, 29]. By differentiating the edited PSCs into intestinal organoids, the authors could show that the mutant organoids have shorter telomeres and fail to maintain budding crypts, resembling diseased phenotypes [118].

### Kidney organoids

The kidney is a highly structured organ consisting of more than 20 cell types, organized into networks of nephrons, vasculature, and interstitial compartments. The establishment of kidney organoids occurs through stepwise-directed differentiation, mimicking the in vivo kidney developmental process [77, 106]. First, 2D cultures of hPSCs are differentiated to intermediate mesodermal cells, then specifically to posterior intermediate mesoderm. Further differentiation steps lead to the establishment of metanephric mesenchyme and later kidney organoids containing nephrons with glomeruli and Bowman’s capsule-like structures, as well as proximal and distal tubules connected by the loop of Henle [77, 106].

In 2015, Freedman and colleagues established a polycystic kidney disease (PKD) model by using CRISPR/Cas9 gene editing to introduce a biallelic mutation in *PKD1* or *PKD2* [31]. The loss of function of either PKD1 or PKD2 is sufficient to induce autosomal dominant polycystic kidney disease whereby affected individuals develop large and numerous renal cysts leading to renal failure [70, 86, 119]. Differentiation of these *PKD1* or *PKD2* mutant hPSCs into kidney organoids led to formation of large renal cysts instead of the tubular nephron-like structures.

## Genetic screening in organoids

Previously, we have discussed the use of CRISPR/Cas9 as an editing tool for genetic modification in organoids. However, recent advances have pushed the limits of editing with large-scale, high-throughput CRISPR/Cas9 screening in organoids. CRISPR/Cas9 screens allow for an unbiased determination of the causal relationship between genotype and phenotype by knocking out gene expression on a genome-wide scale and studying the resulting phenotypic change [18, 38]. In brief, CRISPR/Cas9 screens require an sgRNA library which targets every gene in the genome or in a specific gene set. Multiple sgRNAs are used to target a single gene to reduce false-positives and non-targeting sgRNAs are introduced as negative controls. The sgRNA library can be introduced into the target cells together with Cas9 endonuclease in one combined lentiviral vector or as a two-vector process where sgRNAs and Cas9 are present on separate plasmids. The screen can be performed as an array where each cell culture well is transduced with known sgRNAs from the library. In this way, the resulting phenotype can be immediately associated with a particular sgRNA without requiring sequencing. This method is especially useful for studying phenotype at single-cell level. However, this method is impractical for large-scale screening because of low-efficiency and high cost [1]. Alternatively, CRISPR/Cas9 screens can be performed in a pooled fashion where cells are transduced with bulk sgRNA library at a low multiplicity of infection (MOI) to ensure that cells are not infected by multiple sgRNAs. Infected cells are then selected and put through positive or negative selection for the phenotype of interest. Genome sequencing of selected cells is then performed to identify the sgRNAs of interest [93].

While genome-wide CRISPR/Cas9 screens are widely performed on both human and mouse immortalized cells in two-dimensional cultures [18, 38, 94, 99, 114, 115], technical limitations had limited the ability to perform CRISPR/Cas9 screens in three-dimensional organoids which would provide a more accurate model of the in vivo response. Due to the size of the sgRNA library and inherent noise, large number of cells would be required to have saturated coverage of the targeting sgRNA library in pooled CRISPR/Cas9 screens. The earliest screen performed in organoids targeted a small library of all nine RASGAP genes which function as negative regulators of RAS signaling to determine the relation between RASGAP genes and EGFR-targeted therapy resistance in colorectal cancer [85]. In this study, the authors used electroporation to transfect patient-derived tumor organoids individually with a plasmid containing sgRNAs targeting each of the RASGAP genes and Cas9 endonuclease. Sequencing and mRNA expression levels were used to confirm the introduction of mutation and RASGAP gene knockouts. Using an unbiased CRISPR/Cas9 editing approach with a small-scale target library, Post et al. were able to determine NF1 as the only RASGAP gene which could enhance EGFR resistance growth upon its depletion [85]. In a later study, Planas-Paz et al. [84] performed a screen on biliary epithelial cells-like organoids (BEC-organoids) to determine essential signaling pathway in ductular reaction in which BECs function as facultative liver stem cells in response to injury. The authors utilized a DNA-barcoded lentiviral sgRNA library targeting 192 genes known to be involved in liver regeneration. DNA-barcoding accounts for BEC-organoid heterogeneous growth which could compromise sgRNA representation after selection. Further optimization was also required to determine the number of cells per sgRNA required to compensate for loss of BEC-organoids during passaging and splitting. The authors identified and validated YAP and mTORC1 as important signaling required for ductular reaction [84].

Despite these applications, CRISPR/Cas9 screens were still limited to a selective gene set when performed in organoids as contrast to genome-scale screening in two-dimensional cultures and in order to have a fully unbiased approach, performing a whole genome screening technique in organoids would be most ideal. In March 2020, the first genome-scale CRISPR screening in human intestinal organoid was performed to identify genes inducing resistance to TGF-β signaling [91]. Initial screenings were done by transducing single organoid cell suspension with a library of tumor suppressor genes. The authors found that while organoids could be used to in CRISPR screens, there were issues in limited cell number and heterogenous growth stochastically biasing certain sgRNAs. In order to be able to perform genome-wide screening, Ringel et al. again transduced single organoid cell suspension with the sgRNA library but engineered an approach to analyze sgRNA representation in each single organoid through sequencing as opposed to collecting all surviving organoids for bulk sequencing. This method allows the authors to account for heterogenous growth as organoids would grow clonally from single cells. Using this method, the authors could identify SWI/SNF complex components as regulators of TGF-β activity [91]. In an alternative approach, Michels et al. utilized CRISPR-UMI and a pre-screening method with HepG2 cancer cell lines to account for heterogenous organoid growth and differences in sgRNA functionality within clonal organoids [75]. The authors showed through a preliminary screen with all tumor suppressor genes that pooled screening in organoids is prone to false positives as organoids are notoriously heterogeneous in growth therefore high sgRNA abundance may not be due to a biological effect but due to random outgrowth of any arbitrary organoid. In order to account of this, the authors made use of CRISPR-UMI method where each sgRNA is paired to barcode of ten random nucleotides allowing identification of single cell–derived clones [76]. Furthermore, Michels et al. also showed that sgRNA phenotypic strength and penetrance are more variable in organoids despite prediction from existing design algorithms and functionality in transformed HepG2 cancer cell lines. To improve this, the authors suggest that gRNA-reporter prescreening in cancer cell models could be performed to reduce library size and gain increased coverage. With these modifications, the CRISPR screen was performed with pan-cancer tumor suppressor gene library in APC^−/−^; KRas^G12D^ pre-oncogenic organoids to study clonal advantage in complex microenvironment upon organoid xenotransplantation. The authors could identify TGFBR2 as an essential hit in inducing clonal advantage from the large-scale screen [75].

While CRISPR/Cas9 screening in organoids presents several limitations when compared to cell line screening due to difficulties in manual handling of 3D organoids at a large scale, based on these findings, appropriate scale CRISPR/Cas9 screen is shown to be feasible with organoid models. Initial issues with small cell number for sufficient sgRNA library coverage and heterogenous organoid outgrowth can be solved by single organoid sequencing or transducing large cell population with CRISPR/UMI library. Furthermore, improving sgRNA design for organoids could increase phenotypic induction and penetrance thus enhancing the CRISPR/Cas9 organoid screening platform to eventually allow for targeting patient-specific mutations or vulnerabilities.

## Discussion

In this chapter, we have given examples of the possible applications of different genetic engineering techniques in different types of organoids. It is perhaps helpful to now provide a guideline for the considerations that need to be taken into account when deciding which delivery system and which genetic engineering tools to utilize. Each consideration should be dependent on the type of organoid system to be used, as well as the type and purpose of editing. As a proof of principle, we will discuss two examples to showcase the thought process involved in selecting the necessary tools.

If the aim is to utilize brain organoids to model a certain monogenic disease, one has to consider when the mutation should be introduced and how. As brain organoids are grown from PSCs, it is possible to genetically edit PSCs prior to differentiation into organoids. This method is simpler and more efficient; however, if the desired mutation prevents the progress of the necessary steps involved in the organoid differentiation, then one has to carefully consider the alternative of editing in organoids or utilizing an inducible editing system instead. Secondly, how the disease will be modeled genetically has to be considered. Is constant expression of the mutant sequence necessary? Is a knockdown of gene expression sufficient to generate the disease phenotype? Is the disease caused by a large insertion or deletion mutation or by a point mutation? If only a knockdown of gene expression is sufficient, it would be more efficient to utilize the RNAi system for editing coupled with non-integrating viral transfection. If permanent large insertion is required, using transposons as a method of editing may be effective. However, if a specific single-base pair sequence substitution is the cause of the disease, it would be more precise to utilize the CRISPR/Cas9 system with HDR or base and prime editing for permanent DNA modification.

In another example, if the aim is to use intestinal organoids to conduct a screen for mutations that induce resistance to a specific drug, then different criteria need to be considered. Transposon editing to introduce mutations is generally difficult to perform on the large scale required for this type of screen, in which multiple genes have to be targeted at once. It is therefore more efficient to use the RNAi or CRISPR/Cas9 system. RNAi screens are often conducted with shRNAs rather than siRNAs, as expression of siRNAs is transient and does not leave a molecular signature in the cells they were introduced into, thus making it difficult to analyze the specific effects of each mutation. Furthermore, siRNA libraries are challenging to clone since targeting RNAs need to be arrayed and individually assessed prior to screening. On the other hand, utilizing retro-/lentivirus encoding shRNAs circumvents all these challenges and provides the ability to perform pooled screenings. All shRNAs can be introduced into the same cell culture or organoid dissociates, followed by isolation of single cells for clonal organoid expansion. At the end of the experiment, hits from the screen can be identified by PCR amplification of the shRNA sequence in the genome. However, the main problem with the RNAi system is that it introduces a knockdown rather than a knockout. Knockdowns can lead to hypomorphic changes and if there is insufficient knockdown of the expression, the shRNAs would not be detected as hits in the screen. Furthermore, shRNAs are prone to off-target effects and require consistent expression throughout the experiment for effects to be detected. A more convenient method for use in screens is the CRISPR/Cas9 system. CRISPR/Cas9 editing is precise and can be barcoded, providing an easily identifiable sequence that can be detected during the screen analysis. CRISPR/Cas9 editing is also permanent and still useful for screening in diploid cells.

## Concluding remarks

In this chapter, we have described the various techniques available for genetic manipulation of organoids to use for different purposes. Although it might be tempting to utilize the newest and most challenging techniques to demonstrate the complexity of a study, it is important to consider the requirements, depending on the project. Choosing the techniques and tools with which you are experienced and knowledgeable, and which best support your model and experimental questions, is the most effective way to achieve the desired outcome.
